# Initial Suicide‐Related Disclosure Characteristics, Motivations, and Outcomes Based on Sexual Orientation

**DOI:** 10.1002/jclp.70102

**Published:** 2026-01-31

**Authors:** Veronika Kobrinsky, Brooke A. Ammerman

**Affiliations:** ^1^ Department of Psychology University of Wisconsin‐Madison Madison WI USA

**Keywords:** bisexuality, help‐seeking, self‐disclosure, sexual orientation, suicidal behaviors, suicidal ideation

## Abstract

**Background:**

Self‐disclosure of suicidal thoughts and behaviors (STBs) is integral for risk assessment and intervention. However, limited research elucidates the nuanced characteristics of first disclosure experiences for bisexual individuals, who are disproportionately impacted by suicide yet remain underrepresented in the literature. This study examined the features, motivations, and outcomes of an initial STB disclosure among heterosexual and bisexual individuals.

**Methods:**

Self‐report data from 259 adults (*M*_age_ = 35.40 years; 35.09% bisexual) with a history of STB disclosure recruited through Amazon Mechanical Turk (mTurk) were analyzed with univariate and non‐parametric tests and binary logistic regression models.

**Results:**

Compared to heterosexuals, bisexual participants reported a higher prevalence of suicidal behavior disclosure, seeking formal disclosure recipients, and disclosing through online platforms. They were also more motivated to address physical safety concerns and obtain professional help and more frequently engaged in help‐seeking behaviors post‐disclosure. On average, both groups rated disclosure as helpful, with heterosexual individuals reporting it as more helpful. There was a significant main effect of help‐seeking encouragement from recipients in predicting post‐disclosure help‐seeking engagement.

**Conclusions:**

These findings underscore the necessity of considering the impact of sexual orientation differences in initial STB disclosure processes, which may set the benchmark for subsequent disclosure and help‐seeking trajectories.

**Abbreviations**
CI: Confidence interval
OR: Odds ratio
SM: Sexual minority
STBs: Suicidal thoughts and behaviors

Suicidal thoughts and behaviors (STBs) constitute serious health concerns, with an estimated 726,000 people dying by suicide worldwide each year (World Health Organization 2024). Compared to heterosexuals, sexual minorities (SMs), and particularly bisexual adults, are three to five times more likely to attempt suicide (Hottes et al. 2016), which has been partially attributed to identity‐based stressors, discrimination, rejection, victimization, and delayed healthcare access (Salway et al. 2019; Tabaac et al. 2020; Taylor 2018). Notwithstanding the urgent public health objective of reducing suicide rates among bisexual adults, minimal progress has been made over several decades in the systematic ability to detect STB risk (Franklin et al. 2017), which is a prerequisite for intervention. Communication of STB risk is a key component in facilitating risk assessments and treatment engagement (Michelmore and Hindley 2012; Sheehan et al. 2019). One way to effectively advance intervention efforts is to detect factors contributing to STB disclosure, and lack thereof, among high‐risk SM groups. Although the importance of first‐ever disclosures has been recognized as setting the benchmark for future disclosures and help‐seeking expectancies (Sheehan et al. 2019), research has yet to examine the nuanced characteristics of first disclosure experiences and ensuing help‐seeking outcomes as they relate to bisexuality.

## 1 General STB Disclosure Patterns and Barriers

Evidence suggests that less than half of suicide decedents communicate any history of suicidal ideation or planning to healthcare providers in the year prior to death (Luoma et al. 2002), indicating a critical missed opportunity to employ resources to prevent mortality. In the general population, several barriers to disclosure have been identified, including societal and self‐stigma, shame, fear of rejection and unhelpful resources, perceived lack of risk severity, concerns about involuntary hospitalization, lack of healthcare insurance, and confidentiality concerns (Fulginiti and Frey 2019; Sheehan et al. 2019). Research examining factors that motivate disclosure has found that individuals conduct cost‐benefit analyses prior to sharing STB information, with common motivations being seeking help, strengthening relationships, and sharing personal vulnerability (Blanchard and Farber 2020).

When individuals do choose to disclose STBs, they are often selective about recipients of such communication (Fulginiti et al. 2016; Harris et al. 2009). Studies have indicated that people are generally more inclined to disclose STB history to informal networks, such as friends, compared to medical professionals, who are sometimes perceived as less supportive (Drum et al. 2009). However, the type of STB may influence recipient choice, as individuals with a history of suicide attempts are more likely to present to medical settings and subsequently encounter formal providers who assess risk in a structured manner (Ammerman et al. 2022; Sheehan et al. 2019). Research has also demonstrated differential patterns of disclosure based on patient and interviewer characteristics, with some individuals more likely to conceal suicide intent from clinicians compared to researchers (Bloch‐Elkouby et al. 2023), demonstrating the importance of contextual factors in assessing such communication.

The importance of *first‐eve*r STB disclosures has been recognized as setting the benchmark for future disclosures and help‐seeking expectancies (Sheehan et al. 2019). Drawing on the Disclosure Processes Model, which seeks to explain factors that hinder or motivate the disclosure of concealable health conditions, such as human immunodeficiency virus (HIV), research has found that initiating conversations about personal health information can enhance social support, reduce symptom escalation, and improve adherence to treatment regimens, ultimately bolstering health outcomes (Chaudoir et al. 2011; Smith et al. 2008). Such disclosures provide opportunities to gain health literacy, tangible resources, and positive social experiences that challenge the expectations of stigmatizing interactions (Chaudoir et al. 2011; Mackert et al. 2019). This foundation provides a framework for investigating how *initial* disclosures of STBs might impact treatment seeking and engagement, particularly among minoritized groups, where this remains an understudied area of inquiry.

## 2 Bisexual‐Specific Disclosure Challenges

In contrast to the general population, SMs face additional obstacles in disclosing and seeking help for STBs. These include discrimination, intense fears of prolonged hospitalization and interpersonal negative consequences, and victimization related to both their sexual orientation and mental health concerns (Burke et al. 2021; Hottes et al. 2016; Williams et al. 2018). These additive stressors may lead to distinct considerations when evaluating whether to communicate about suicide risk. Given that nondisclosure has been linked to increased distress (Levi‐Belz et al. 2019), social isolation (Husky et al. 2016), greater lethality of suicide attempts (Trakhtenbrot et al. 2016), unmet personal needs, negative help‐seeking attitudes, and higher mortality rates (Hogge and Blankenship 2020; Luoma et al. 2002), understanding disclosure patterns among SMs is particularly critical.

A poorly addressed gap in research considering demographic differences in first STB disclosures has been the historical conceptualization of within‐SM identities as homogeneous and qualitatively similar (Diamond 2008). Consequently, the extant literature predominantly reports on SM data in aggregate, which obscures the role that unique bisexual‐specific characteristics have in the STB disclosure and help‐seeking process (Clark and Blosnich 2023; Hatchel et al. 2021; Meyer et al. 2015), raising an area of concern as SMs are not monolithic groups and have disparate suicide outcomes (Haas et al. 2011; Salway et al. 2019).

Supported by the minority stress model (Meyer 2003), bisexual individuals face unique barriers to communicating their risk as a result of “double stigma” from both within the SM community and society at large. These experiences include homosexism, stereotype threat, bisexual erasure, social invisibility, internalized binegativity, and a lack of bisexual‐affirming resources (Chang et al. 2022; Salway et al. 2019). These structural and interpersonal disparities disincentivize the disclosure of both sexual identity and STBs, further exacerbating social alienation, avoidance of the healthcare system, and suicide risk (Casey et al. 2019). Meta‐analytic research has confirmed that compared to heterosexual and lesbian or gay respondents, bisexual individuals report the highest proportion of suicide ideation and attempts but are unlikely to discuss this with healthcare providers and informal networks (Salway et al. 2019).

## 3 Disclosure Recipients and Modality Considerations for Bisexual Individuals

Important differences may exist between bisexual and heterosexual individuals in their choice of disclosure recipients. Bisexual individuals hold a socially stigmatized position and generally report greater interpersonal stressors and more limited social support networks (Meyer 2003; Hatchel et al. 2021). These factors may limit their access to trusted informal supports for STB disclosure. While bisexual individuals appear to have greater healthcare discrimination and fewer intentions to engage with mental healthcare than monosexual individuals (DeLucia and Smith 2021; Doan Van et al. 2019), the higher rates of STBs observed in this group may predispose them to presenting in emergency settings, thereby resulting in subsequent STB disclosure to healthcare providers.

Disclosure modality preferences may also be influenced by stigma and minority stress experiences. Research with marginalized groups has shown a preference for disclosing mental health problems through social media communities, which offer increased anonymity, security, and safety (Ammerman et al. 2025; Berry et al. 2017; O'Dea et al. 2017). While online discussions of mental health and suicide have become more prevalent, there is limited research examining how bisexual individuals choose between different disclosure modalities when sharing STB experiences. Data from university students has demonstrated a preference for communicating about and seeking help for STBs online rather than face‐to‐face, possibly due to the reduced stigma associated with discussing suicide through digital platforms for this age group (Wilks et al.^2019^ ). This digital preference observed in college‐aged students, who represent one group with high suicide rates and multiple interacting barriers to mental health care service utilization (Horwitz et al. 2020), may also be relevant to bisexual individuals who experience similar complex obstacles to accessing healthcare. Internet‐based platforms may provide several advantages for stigmatized populations, such as bisexual individuals, including anonymity, access to specialized communities, and decreased fear of immediate negative consequences. Considering the intertwined minority stressors experienced by bisexual communities, internet‐based platforms for disclosure and intervention may help mitigate attitudinal and practice barriers that exist in traditional healthcare settings (Ren et al. 2022). However, more research is needed to identify which modalities bisexual individuals are most likely to utilize when sharing information about their STBs.

## 4 Current Study

The main objective of the current research was to extend the knowledge of STB disclosure characteristics by examining potential differences in motivations, recipient choices, modality, and help‐seeking outcomes of an initial disclosure between heterosexual and bisexual adults. Based on the existing literature, we predicted that bisexual individuals will (1) report higher rates of suicidal behaviors and subsequent disclosures of those behaviors compared to heterosexual individuals (Hottes et al. 2016), (2) be more likely to disclose to formal healthcare providers rather than informal supports, given their higher rates of severe STBs and potential emergency department presentations (Salway et al. 2019), (3) prefer virtual disclosure modalities over face‐to‐face communication (Ammerman et al. 2025), (4) be more motivated by physical safety concerns and professional help‐seeking when making disclosure decisions, reflecting the severity of STB experiences commonly observed in this population (Salway et al. 2019), (5) rate disclosure as less helpful than heterosexual individuals, given potentially increased rates of interactions with emergency healthcare systems (Guzmán et al. 2020), and (6) engage in help‐seeking behaviors at lower rates following disclosure, considering the pervasive discrimination and inequitable healthcare experiences reported by this population (Calear et al. 2014). No a priori hypotheses were made regarding motivations to obtain emotional support, closer relationships with recipients, and achieve stigma‐related reduction goals due to the scarcity of research in this domain.

## 5 Methods

### 5.1 Participants and Procedures

Participants were recruited from Amazon Mechanical Turk (mTurk), a crowdsourcing marketplace previously used in studies examining clinical characteristics (Arditte et al. 2016). Inclusion criteria included age 18 years or older, location within the United States, a 95% or higher mTurk approval rate, at least one lifetime episode of STBs, and at least one prior STB disclosure. All study procedures were approved by the senior author's former Institutional Review Board (IRB #20‐07‐6104; “A Research Study on the Self‐Disclosure of Suicidal Thoughts and Behaviors”) and informed consent was obtained from all participants. Consenting participants completed a battery of online questionnaires and were compensated for their time. To be included in the current analyses, participants had to endorse at least one prior STB disclosure, pass an online bot‐detection item (i.e., ReCaptcha), and respond to at least 50% of eight attention check items correctly (i.e., “Please select “1” for this item”).

The study sample consisted of 259 adults (*M*_age_ = 35.40 years, SD = 10.08, range = 19–68). There were 169 (65.30%) participants identifying as male, 85 (32.80%) as female, 2 (0.80%) as transgender, 2 (0.80%) as nonbinary, and 1 (0.40%) person did not report gender. Overall, 64.91% of the sample identified as heterosexual and 35.09% as bisexual. The majority of the sample was White (70.30%), followed by Black or African American (25.10%), Asian (6.60%), Alaska Native (2.70%), or another race (1.20%). Less than half of the sample (30.50%) reported a Hispanic/Latine ethnic identity.

## 6 Measures

### 6.1 Suicidal Thought and Behavior History

Three face‐valid, dichotomous questions were used to assess lifetime STB history. Suicidal ideation was examined by asking participants, “Have you ever thought of killing yourself,” suicide planning with the question, “Have you ever had a plan to kill yourself?”, and the third question assessed lifetime suicide attempt, “Have you ever made an actual attempt to kill yourself in which you had at least some intent to die?”.

### 6.2 Suicidal Thought and Behavior Disclosure Experiences

Study eligibility included an endorsement of the item, “Have you ever told another person, whether face‐to‐face or online, about your thoughts or attempts to kill yourself?”. To assess details of initial disclosure experiences, participants were asked several items examining whether they disclosed or concealed a history of suicidal behaviors, disclosure recipient, and modality. Participants were categorized into two disclosure groups based on the severity of STBs disclosed during their first disclosure experience. The “suicidal ideation disclosure group” included individuals who disclosed only suicidal thoughts during initial disclosure, regardless of whether they had engaged in but concealed more severe behaviors (i.e., planning or attempts). The “suicide behavior disclosure group” included individuals who disclosed any suicide planning or attempt behaviors during their first disclosure. This distinction allowed us to examine differences based on the severity level of STBs that participants chose to share. Due to small cell sizes in specific recipient and modality categories, responses were recoded into broader, theoretically meaningful groups. Disclosure recipients were recoded as “informal recipients” (including friends, significant others, immediate and extended family members, and online acquaintances such as those met via Reddit) versus “formal recipients” (including medical professionals, mental health professionals, crisis providers such as hotlines, and other professionals such as police or emergency medical technicians). Disclosure modality was recoded as “in‐person” versus “virtual” communication. The virtual category included video calls, phone calls, text messages, online forums/chat, and email, while the in‐person category included only direct face‐to‐face disclosure. This recoding approach allowed for meaningful statistical comparisons while maintaining theoretical distinctions between personal and professional relationships, as well as direct versus digital communication. Eight disclosure motivations were ranked in order of importance from 1 = *Most important reason you disclosed* to 8 = *Least important reason you disclosed*; lower scores indicate greater importance. Participants were also asked to rate the helpfulness of disclosure on a scale from 1 = *Very unhelpful* to 6 = *Very helpful*. Help‐seeking encouragement from recipients and actual engagement in help‐seeking were evaluated with two binary responses (0 = *No*, 1 = *Yes*). See Appendix A for the full list of items and response options.

### 6.3 Data Analyses

Analyses were conducted with SPSS 29.0. Missing data ranged from 0% to 8.5% (i.e., informal and formal recipients) across all variables, with most key variables having less than 2% of missing data. We conducted Little's Missing Completely at Random (MCAR) test to evaluate the pattern of missingness. The results indicated that data were missing completely at random (*χ*² = 23.45, *df* = 39, *p* = 0.89), informing the use of listwise deletion to handle missing values. An analysis of skew suggested that variables were not significantly skewed (range = −0.13 to 1.21) and kurtosis was within an appropriate range (−1.23 to 0.95), meeting normality assumptions. Descriptive statistics were used to examine the general prevalence of STBs and disclosure characteristics. Univariate (*t*‐tests and chi‐square, ***χ*** ^2^) and non‐parametric (Mann‐Whitney *U*) tests were used to examine differences in disclosure motivations, characteristics, and outcomes between heterosexual and bisexual individuals. Mann‐Whitney *U* tests were used for the ranked disclosure motivation variables as these items created ordinal data, which do not meet the interval assumptions of parametric tests. Mean ranks for the Mann‐Whitney *U* are calculated by ranking the data for each sexual orientation group which allows for a comparison of how different the two rank totals are. In the current study, STB disclosure motivations were ranked such that lower scores indicated greater importance; therefore, lower mean ranks for each motivation demonstrate a higher importance for disclosing STBs. Cramer's *V* and *z*‐scores are presented wherever appropriate to aid in interpreting results. To assess the influence of help‐seeking encouragement on help‐seeking behaviors, a binary logistic regression was conducted in which an interaction term (i.e., help‐seeking encouragement*sexual orientation) was created to test the influence of sexual identity on behavioral outcomes. The regression model result is presented as an odds ratio (OR) with a 95% confidence interval (CI). The current study is among the first to examine initial disclosure characteristics among a within‐SM group; therefore, for purposes of replication with larger and more diverse samples, a critical alpha of 0.05 was used.

## 7 Results

### 7.1 Preliminary Analyses

Of the entire sample, 248 (95.80%) individuals had a history of suicidal ideation, 150 (57.90%) had previously made a suicide plan, and 138 (53.30%) had at least one prior suicide attempt. The rates of suicidal ideation between heterosexual (96.32%) and bisexual (94.32%) participants were not significantly different, *χ* ^2^ = 0.55, *df* = 1, *p* = 0.46. Bisexual participants endorsed significantly greater frequencies of suicide planning (72.73%) and suicide attempts (71.59%) compared to heterosexuals (50.31%; *χ* ^2^ = 11.81, *df* = 1, *p* < 0.01, and 43.56%; *χ* ^2^ = 18.05, *df* = 1, *p* < 0.01, respectively). A total of 103 (39.80%) participants disclosed only suicidal ideation, 56 (21.60%) disclosed suicidal ideation but concealed a suicide plan and/or attempt, 49 (18.90%) had disclosed a suicide plan, 12 (4.60%) disclosed a suicide plan but concealed a suicide attempt, and 39 (15.10%) disclosed only a suicide attempt.

### 7.2 General Disclosure Characteristics

Chi‐square and independent samples *t*‐tests indicated that there were significant differences in general disclosure characteristics based on sexual orientation. Namely, compared to heterosexual participants, bisexuals had greater rates of suicidal behavior disclosures (*χ* ^2^ = 12.94, df = 1, *p* < 0.01), disclosing to formal healthcare professionals rather than informal supports (*χ* ^2^ = 10.35, df = 1, *p* < 0.01), and disclosing through virtual platforms as opposed to face‐to‐face (*χ* ^2^ = 10.36, *df* = 1, *p* < 0.01; see Table 1). Heterosexual participants (*M* = 4.77, SD = 1.35) rated their disclosure experiences as more helpful, on average, than bisexual individuals (*M* = 4.70, SD = 1.00), *t* = 0.44, df = 229, *p* < 0.01.

**Table 1:** STB disclosure and help‐seeking characteristics by sexual orientation.

	Heterosexual	Bisexual			
	*N* (%)	*N* (%)	*χ*2	*p*‐value	Cramer's *v*
**STB disclosure type**			**12.94**	< 0.01	0.23
Suicidal ideation	112 (68.71%)	40 (45.45%)			
Suicidal behavior	51 (31.29%)	48 (54.55%)			
**Disclosure recipient**			**10.35**	< 0.01	0.20
Informal	135 (82.82%)	57 (64.77%)			
Formal	28 (17.18%)	31 (35.23%)			
**Disclosure modality**			**10.36**	< 0.01	0.20
In‐Person	103 (63.19%)	37 (42.05%)			
Virtual	60 (36.81%)	51 (57.95%)			
**Help‐seeking encouragement**			**6.40**	0.01	0.17
No	64 (42.11%)	19 (25.00%)			
Yes	88 (57.89%)	57 (75.00%)			
**Help‐seeking engagement**			**14.82**	< 0.01	0.26
No	95 (62.50%)	27 (35.53%)			
Yes	57 (37.50%)	49 (64.47%)			

*Note:* Significant chi‐square values are presented in bolded font.

### 7.3 Disclosure Motivations

Significant differences emerged between both groups in evaluating the motivations underlying initial STB disclosures. Bisexual participants were more likely to disclose the presence of STBs due to physical safety concerns, *U* = 6075.00, *p* = 0.04, and wanting to seek professional help, *U* = 4893.00, *p* < 0.01. On the other hand, heterosexual individuals rated a higher perceived obligation to disclose their STBs, *U* = 4395.50, *p* < 0.01. There were no significant differences in motivations of emotional support, wanting a closer relationship with a disclosure recipient, perceived pressure to disclose, and stigma reduction (see Table 2).

**Table 2:** STB disclosure motivations by sexual orientation.

	Heterosexual	Bisexual			
	Mean rank	Mean rank	Mann‐Whitney U	*z*‐score	*p*‐value
Emotional support	125.00	125.01	7083.50	−0.01	0.98
Professional help	138.61	100.10	4893.00	**−4.08**	< 0.01
Physical safety	131.27	113.53	6075.00	**−1.90**	0.04
Closer relationship with recipient	123.80	127.20	6890.50	−0.37	0.72
Pressure to disclose	129.13	117.44	6418.50	−1.25	0.21
Stigma reduction	121.98	130.52	6598.00	−0.91	0.36
Perceived obligation to disclose	108.30	155.55	4395.50	**−5.05**	< 0.01

*Note:* Significant *z*‐scores are presented in bold font.

### 7.4 Help‐Seeking Encouragement and Behavior

In examining the impact of disclosure on help‐seeking encouragement and behaviors, bisexual individuals reported receiving greater help‐seeking encouragement from recipients of disclosure, χ2 = 6.40, df = 1, *p* = 0.01, and they sought help following disclosure more frequently than heterosexual participants, χ2 = 14.82, df = 1, *p* < 0.01 (Table 1). A binary logistic regression was used to investigate whether encouragement to seek help was predictive of actual help‐seeking behaviors and whether sexual orientation influenced this relationship. As demonstrated in Table 3, there was a significant main effect of help‐seeking encouragement on actual behaviors following disclosure, and there was no influence of sexual orientation on this relationship.

**Table 3:** Help‐seeking encouragement from recipients predicting healthcare engagement.

Predictor	Help‐Seeking Behavior		
	OR	95% CI	*p*
Sexual Orientation	2.58	0.64, 4.52	0.18
Help‐Seeking Encouragement	13.32**	7.20, 15.12	< 0.01
Help‐Seeking Encouragement*Sexual Orientation	1.06	0.22, 5.14	0.95

*Note:* *p < 0.05; **p < 0.01; Significant odds ratios (ORs) are presented in bold font.

## 8 Discussion

The present study is among the first to investigate the multifaceted characteristics of first STB disclosure experiences, including motivations, recipient choices, modality, and help‐seeking outcomes, among bisexual and heterosexual individuals. Our first hypothesis predicted that bisexual individuals would report higher rates of suicidal behaviors and subsequent disclosure of those behaviors compared to heterosexual individuals. This was supported as bisexual participants endorsed significantly greater frequencies of suicide planning (72.73% vs. 50.31%) and suicide attempts (71.59% vs. 43.56%) compared to heterosexuals and were more likely to disclose suicidal behaviors rather than ideation only. These findings align with previous meta‐analytic research demonstrating that bisexual individuals report the highest proportion of suicidal behaviors compared to heterosexual and lesbian/gay counterparts (Salway et al. 2019). These higher disclosure rates of behaviors, versus ideation alone, among bisexual participants may reflect the greater severity of their STB experiences, which often necessitate disclosure in crisis or medical settings.

Our second hypothesis predicted that bisexual individuals would be more likely to disclose to formal healthcare providers rather than informal supports. This hypothesis was supported as bisexual participants had significantly greater rates of disclosing to formal healthcare professionals rather than informal supports compared to heterosexual participants. These findings extend previous literature indicating that people who attempt suicide are vulnerable to coming into contact with the healthcare system and may have streamlined exposure to healthcare providers who assess for suicide risk (Ammerman et al. 2022; Sheehan et al. 2019). The greater perceived severity of suicide attempts may be one factor contributing to bisexual participants' more frequent involvement with healthcare providers. Additionally, bisexual individuals hold a socially stigmatized position in society and generally experience greater interpersonal stressors. These social challenges may result in more constrained social support networks (Meyer 2003; Hatchel et al. 2021), potentially limiting their access to trusted informal supports for STB disclosure.

Third, we predicted that bisexual individuals would prefer virtual disclosure modalities over face‐to‐face communication, which was supported by the data. Bisexual participants were significantly more likely to disclose through virtual platforms as opposed to face‐to‐face communication. With the existence of dual stigma concerning bisexuality and suicidality (Chang et al. 2022; Sheehan et al. 2019), online platforms likely provide additional anonymity, community support, and decreased fear of immediate negative consequences following disclosure. This finding supports meta‐analytic research showing that disclosure is more prevalent when information is exchanged online compared to in person (Hallford et al. 2023). Online platforms may be particularly helpful for bisexual individuals by reducing the barriers that are present in traditional healthcare settings. Thus, it is important to improve and understand the efficacy of digital‐based platforms, such as the Trevor Project (Phillips 2022) and Crisis Text Line (Pisani et al. 2022), in which trained gatekeepers support and connect individuals with crisis resources. Such interventions may augment methods for reaching high‐risk communities that may otherwise go undetected by traditional risk assessments (Frey et al. 2016). Online support groups that are tailored to the needs of bisexual individuals can also serve as safe spaces that increase bisexual visibility and challenge oppressive social dynamics which hinder the disclosure of sensitive topics (Maliepaard 2017). Concerns have been raised that online communities could be a source of potentially negative and explicit STB‐related content (Lewis et al. 2011); however, preliminary evidence supports the importance of online support for the destigmatization of STBs and a sense of belonging for people who might lack off‐line resources (Whitlock et al. 2006).

While our findings demonstrate that bisexual individuals significantly preferred virtual disclosure modalities over face‐to‐face communication, it is important to acknowledge that our operationalization of virtual disclosure encompassed diverse formats including video calls, phone calls, text messages, online forums/chats, and email that were collapsed into broader categories. Although this inclusive categorization was practical for statistical analysis and aligns with prior research distinguishing in‐person and digital communication channels (Seward and Harris 2016; Wong et al. 2021), it potentially obscures meaningful distinctions between these modalities and their potential impact. For instance, anonymous participation in online forums led by individuals with lived experiences, such as Reddit communities, may offer substantially different disclosure opportunities compared to video telehealth sessions with identities healthcare providers (Harris et al. 2009).

Anonymous platforms may maximize perceived safety through complete identity concealment and reduced fear of interpersonal consequences, while video‐based platforms retain many features of face‐to‐face interaction, including visual presence and real‐time responsiveness, yet still offer geographical accessibility and reduced logistical barriers compared to in‐person healthcare visits (Gordon et al. 2023). Text‐based asynchronous communication (e.g., email, text messaging) provides additional time for reflection and careful message construction, which may be particularly valuable when navigating the dual stigma associated with bisexuality and suicide (Chang et al. 2022; Sheehan et al. 2019). Future research would benefit from disaggregating these virtual modalities to examine whether specific communication methods differentially predict disclosure motivations, recipient selections, perceived helpfulness, and subsequent help‐seeking engagement, particularly among bisexual individuals who may strategically select platforms based on anonymity affordances, community availability, and anticipated discrimination. Understanding these nuanced perspectives would enable more targeted development and dissemination of digital mental health resources tailored to the specific needs and concerns of bisexual communities.

Bisexual individuals were also predicted to have greater motivations to disclose due to physical safety concerns and professional help‐seeking intentions when making disclosure decisions. This was partially supported in that bisexual participants were significantly more likely to disclose STBs due to concerns about their ability to keep themselves safe and wanting professional intervention compared to heterosexual individuals. In other words, when prioritizing their reasons for disclosure, bisexual individuals placed higher importance on these safety‐related and professional intervention motivations. However, contrary to our prediction, heterosexual individuals ranked perceived obligations to disclose as a more important motivation than did bisexual participants. This finding may reflect that heterosexual individuals experience fewer identity‐related stressors and may not face the same potential for social exclusion when seeking mental health resources (Corrigan et al. 2012). Bisexual individuals might feel less perceived disclosure obligation due to stigma‐related mechanisms such as expectations of rejection, discrimination, and internalized biphobia (DeLucia and Smith 2021; Meyer 2003).

We found that bisexual participants rated their initial disclosure experiences as less helpful than heterosexual individuals, supporting our fifth hypothesis. This finding may reflect that bisexual individuals engaged with healthcare professionals more commonly, and research suggests that healthcare providers may be perceived as less supportive when discussing suicide risk (Drum et al. 2009), compared to mental health professionals trained in managing STBs. Additionally, bisexual individuals often report lack of sexual‐orientation affirming healthcare and high rates of healthcare discrimination (Tabaac et al. 2020), which may have impacted their help‐seeking experiences. Importantly, both groups rated their disclosure experiences as overall helpful (with means above a 4 on a 1–6 scale), suggesting that in this sample, disclosure generally served a positive first step toward intervention.

Finally, contrary to our predictions, bisexual individuals reported receiving greater help‐seeking encouragement from recipients and sought help following disclosure more frequently than heterosexual participants (64.47% vs. 37.50%). This finding suggests that despite potentially experiencing healthcare discrimination, the severity of bisexual individuals' STB experiences and their disclosure to formal providers might have facilitated more active help‐seeking engagement. The Behavioral Model of Health Services Use (Andersen 2008) proposes that external reinforcement is a key determinant of help‐seeking behaviors, and our findings support that encouragement to utilize available resources from recipients significantly predicted actual post‐disclosure healthcare decisions across both groups. Positive disclosure experiences can provide people with a pathway for accessing professional services and social support (Sheehan et al. 2019), and more research is warranted to understand the factors constituting positive or negative initial disclosures.

### 8.1 Limitations

The present findings should be considered in light of several methodological limitations. Our sample was demographically biased as the majority of participants identified as white and female. There is a need to more clearly understand the influence of intersectional sociodemographic characteristics in the decisions, motivations, and outcomes of initial STB disclosures. Inclusion criteria for the current study required that participants have at least one prior disclosure which obscures the prevalence of nondisclosure. Furthermore, although mTurk is a common platform for recruiting participants to examine clinical characteristics (Arditte et al. 2016), this study will need to be replicated with traditional clinical and community samples to be representative of individuals who might be at highest risk for STBs. An important limitation concerns our assessment of partial disclosure patterns. While capturing instances where individuals disclose suicidal ideation but conceal more severe experiences like planning or attempts reflects important real‐world disclosure dynamics that have been understudied, the measurement approach used in this study has not been validated. Additionally, we have only focused on initial disclosures, and it will be important to assess a broader spectrum of help‐seeking behaviors. Future research should prioritize developing and validating more comprehensive measures that can reliably assess diverse disclosure patterns.

## 9 Conclusions

The current study holds immediate research and clinical implications. The results demonstrate key differences in initial STB disclosure processes between heterosexual and bisexual individuals. The findings underscore the importance of tailoring suicide prevention and assessment strategies to address the unique needs of bisexual adults, who were more likely to disclose suicidal behaviors, safety concerns, and desire to receive professional support, as well as use online platforms for disclosure. Researchers and clinicians are encouraged to adapt suicide risk assessments to include the examination of potential risk factors that are contextually relevant for people holding socially stigmatized identities, such as the presence of discrimination, victimization, healthcare inequity, and bisexual‐specific stressors. Furthermore, rapport can be enhanced through communication that emphasizes inclusivity, safety, and explicit acknowledgment of structural factors impacting the lives of bisexual adults. The encouragement to seek support from disclosure recipients also played a critical role in promoting actual help‐seeking behaviors across sexual orientation groups. Specifically promoting online resources and support networks can provide bisexual individuals with opportunities for disclosure and community building. Overall, communicating about STBs was rated as helpful, and future research and clinical work should focus on investigating the fine‐grained details of positive disclosure experiences that can promote help‐seeking behaviors.

## Ethics Statement

All study procedures were conducted in accordance with the ethical standards of the 1964 Declaration of Helsinki and its later amendments and were approved by the senior author's former Institutional Review Board.

## Supporting information

SDT DP1 Appendix A.

## Data Availability

The data are available on request from the authors.
