# Supplementary material for: Initial Suicide‐Related Disclosure Characteristics, Motivations, and Outcomes Based on Sexual Orientation
Source: J Clin Psychol. 2026 Jan 31;82(5):701–9. doi: 10.1002/jclp.70102 (PMC13033373; doi:10.1002/jclp.70102)
Supplement: Supplementary file 1 — SDT DP1 Appendix A. [file JCLP-82-701-s001.docx]

**Appendix A: Suicidal Thought and Behavior Disclosure Experiences**

Which of the following experiences did you disclose at this time? If you disclosed multiple experiences, please report the most serious experience.

1. Suicidal ideation only.
2. Suicidal ideation only, but I have had a suicide plan or attempted suicide (but did not disclose this).
3. Suicide plan only.
4. Suicide plan only, but I have had attempted suicide (but did not disclose this).
5. Suicide attempt.

Who did you first disclose your suicidal thoughts and/or behavior to?

1. Friend.
2. Significant other/spouse.
3. Other immediate family member.
4. Extended family member.
5. Medical professional (i.e., primary care provider, psychiatrist, emergency room staff).
6. Mental health professional (i.e., counselor, therapist, psychologist).
7. Someone/others you only knew only (e.g., via Reddit).
8. Crisis providers (i.e., National Suicide Hotline, Crisis Text Line).
9. Other professionals (i.e., police officers, social worker, EMT).
10. Other: ___________

How did you first disclose your suicidal thoughts and/or behavior to this person?

1. In-person.
2. Via video call (i.e., FaceTime, Zoom, Skype).
3. Phone call without video.
4. Text message.
5. Online forum, chat, or instant messenger.
6. Email.
7. Letter.

Why did you disclose at this time? Please rank in order of importance by assigning each potential reason a number 1 through 8, where 1 = most important reason you disclosed and 8 = least important reason you disclosed.

- I wanted to receive emotional support.
- I wanted to get professional help.
- I wanted help maintaining my physical safety (i.e., was afraid I would act on suicidal thoughts).
- I wanted to feel closer to the person I disclosed to.
- I felt pressured to disclose because of the situation (i.e., due to being at the emergency room, welfare check, etc.).
- I wanted to help reduce stigma or misconceptions about suicidal thoughts or behavior.
- I felt obligated to tell someone (i.e., felt like I was “lying” or being dishonest by withholding this information from someone close to me).
- Other: ___________

Overall, how would you rate this disclosure experience on the following:

- Helpfulness (1 - 6: 1 = Very Unhelpful, 6 = Very Helpful).

Did this person encourage you to seek professional help (i.e., see a counselor or therapist, go to the emergency room, seek hospitalization)?

0. No.

1. Yes.

Did you seek professional help as a result of this disclosure?

0. No.

1. Yes.
